# Skeletal Muscle, Skin, and Bone as Three Major Nitrate Reservoirs in Mammals: Chemiluminescence and ^15^N-Tracer Studies in Yorkshire Pigs

**DOI:** 10.3390/nu16162674

**Published:** 2024-08-13

**Authors:** Barbora Piknova, Ji Won Park, Khalid J. Tunau-Spencer, Audrey Jenkins, David G. Hellinga, Peter J. Walter, Hongyi Cai, Alan N. Schechter

**Affiliations:** 1 National Institute of Diabetes and Digestive and Kidney Diseases (NIDDK), National Institutes of Health (NIH), Bethesda, MD 20892, USA; 2 MedStar Health Research Institute, Washington, DC 20010, USA; 3 Clinical Mass Spectrometry Core, National Institutes of Health (NIH), Bethesda, MD 20892, USA

**Keywords:** skeletal muscle nitrate reservoir, nitric oxide reductive pathway, nitrate in skin and bone

## Abstract

In mammals, nitric oxide (NO) is generated either by the nitric oxide synthase (NOS) enzymes from arginine or by the reduction of nitrate to nitrite by tissue xanthine oxidoreductase (XOR) and the microbiome and further reducing nitrite to NO by XOR or several heme proteins. Previously, we reported that skeletal muscle acts as a large nitrate reservoir in mammals, and this nitrate reservoir is systemically, as well as locally, used to generate nitrite and NO. Here, we report identifying two additional nitrate storage organs—bone and skin. We used bolus of ingested ^15^N-labeled nitrate to trace its short-term fluxes and distribution among organs. At baseline conditions, the nitrate concentration in femur bone samples was 96 ± 63 nmol/g, scalp skin 56 ± 22 nmol/g, with gluteus muscle at 57 ± 39 nmol/g. In comparison, plasma and liver contained 34 ± 19 nmol/g and 15 ± 5 nmol/g of nitrate, respectively. Three hours after ^15^N-nitrate ingestion, its concentration significantly increased in all organs, exceeding the baseline levels in plasma, skin, bone, skeletal muscle, and in liver 5-, 2.4-, 2.4-, 2.1-, and 2-fold, respectively. As expected, nitrate reduction into nitrite was highest in liver but also substantial in skin and skeletal muscle, followed by the distribution of ^15^N-labeled nitrite. We believe that these results underline the major roles played by skeletal muscle, skin, and bone, the three largest organs in mammals, in maintaining NO homeostasis, especially via the nitrate–nitrite–NO pathway.

## 1. Introduction

Several decades after the identification of NO as an endothelium-derived relaxing factor (EDRF) [1], we are still discovering the importance of and new roles played by this small gaseous molecule in normal physiology, as well as the pathophysiology of many unrelated diseases [2]. Soon after the discovery of the role of NO in vasorelaxation in the 1980s, a family of NO-producing enzymes was identified and named nitric oxide synthase (NOS), with three currently known isoforms: NOS1 (nNOS, neuronal NOS), NOS2 (iNOS, inducible NOS) and NOS3 (eNOS, endothelial NOS). All three isoforms synthetize NO in the reaction of L-Arg with oxygen, leading to NO and L-citrulline (for a current review, see [3]). There are severe health consequences of inadequate NO being supplied by these NOS isoforms, and, interestingly, they depend on the missing NOS isoform [4]. There is a parallel pathway where NO is the product of nitrite reduction by heme- and molybdenum-containing proteins [5,6,7], and nitrite itself is the product of nitrate reduction by symbiotic bacteria or molybdenum-containing mammalian proteins [8,9]. The efficiency of this reductive NO pathway (nitrate–nitrite–NO) increases inversely proportional to oxygen content [10,11,12,13]. This way, to ensure adequate NO supply, both pathways are running simultaneously with different efficiencies depending on local tissue oxygenation. 

One can consider nitrate and nitrite as NO precursors with different reactivities and lifetimes [11,14]. In mammals, diet is the main nitrate source, together with the oxidation of nitrite and NO by oxyheme proteins [11]. Nitrate is largely stable in blood and body tissues, with salivary/gut microbiome appearing to provide the largest volume of the reduction of nitrate into nitrite [8,15,16,17,18,19,20,21]. Mammalian endogenous nitrate reductases—molybdopterin-containing mammalian proteins, such as xanthine oxidoreductase (XOR) [9,22,23], aldehyde oxidase (AO) [5,7], sulfite oxidase (SO) [24], and mitochondrial amidoxime reducing component (mARC)—also provide additional nitrate reduction into nitrite in hypoxic tissues [5,25]. The reduction of nitrate into nitrite is thought to be the main source of nitrite in mammals [11,12], but more research is needed to elucidate the relative contribution of NOS-derived and reductive pathways [26]. Nitrite is the direct precursor of NO and, in tissues with decreased oxygen content, it can be reduced to NO by heme- and molybdenum-containing proteins [6,7,9,12,27,28,29,30,31]. Several studies on animals and humans have shown the beneficial effect of a nitrate-rich diet on cardiovascular and metabolic health [11,32,33,34,35], as well as for increasing athletic performances [20,36,37,38,39,40,41,42,43]. 

Previously, based on our extensive studies on rodents and pigs, we found that nitrate is distributed among tissues in a nonuniform way. We found that skeletal muscle tissue contained elevated concentrations of nitrate compared to most other internal organs, forming a renewable nitrate reservoir, and there was a distinct concentration gradient of nitrate from muscle to plasma and liver [44]. We later confirmed this hypothesis on healthy human volunteers [45]. Based on these studies, we formulated the hypothesis of skeletal muscle as a main nitrate reservoir and regulator of systemic NO homeostasis [46]. We showed that dietary nitrate supplementation is an effective way to increase the skeletal muscle nitrate reservoir [47,48]. We also proposed that blood serves as a main transport media for nitrate from skeletal muscle to liver, where nitrate is reduced into nitrite and nitrite is then distributed back to tissues, where it is locally reduced into NO upon demand [49,50,51,52]. It is important to keep in mind that tissues are also able to process nitrate and nitrite locally using their nitrate/nitrite reductases, so the immediate local NO demand can be satisfied in several different ways [44,48,52]. 

While the role of nitrate/nitrite in NO formation in skeletal muscle is a relatively new concept, there had been a substantial interest in the presence and functions of NO in the skin since the early 2000s. As early as in 1996, Benjamin and colleagues hypothesized that NO is generated on the skin surface from sweat nitrate upon UVA (ultraviolet A) irradiation [53]. Several groups showed that skin-derived NO originates from the photolysis of S-nitrosothiol-proteins (SNO-proteins) and nitrite present in the skin [54,55,56]. Physiological effects of NO generated in the skin included lowering blood pressure [57,58], increasing cutaneous blood flow [59], and wound healing [60,61,62,63]. The main NO-active (precursor) components had been established as SNO-proteins and nitrite, both susceptible to photolysis, and effects had been described as transient, i.e., present shortly after nitrate supplementation [59,64]. Recently, the microdialysis of interstitial fluid in the skin showed lower nitrate but higher nitrite content in the skin’s interstitial fluid at baseline, when compared to plasma, and a significant increase in both, nitrate and nitrite, after a dietary nitrate supplementation in plasma as well as in the skin’s interstitial fluid [65]. 

The role of NO in bone metabolism had been studied for the past two decades, with much emphasis on osteoporosis and fracture prevention and healing [66,67,68,69,70,71,72,73,74,75,76,77,78]. Through its signaling pathways, NO enhances bone fracture healing [69,74,77]. Although the research on its preventive effect on osteoporosis is still inconclusive, there is a long list of studies showing at least some positive benefits [66,67,74,76], especially in regulating bone blood flow [70,77,79]. Recently, an extensive review summarized the nutritional guidelines for optimal musculoskeletal health and emphasized the adequate intake of dietary nitrate as one of the important factors in the prevention of fall-associated hip fracture, especially in older adults [78]. 

In the present study, we further inquired about the possibility that, in addition to skeletal muscle, some other large organs and tissues also serve as nitrate reservoirs. We identified skin and skeleton as supplementary tissues/organs in pig that store nitrate. Also, using ^15^N-labeled dietary nitrate, we were able to follow short-term flows of nitrate from the blood into organs and identify organs that rapidly (within 3 h past ingestion) sequestered newly supplied nitrate. Surprisingly, levels of ^15^N-nitrate from the blood increased the fastest in skin. The use of ^15^N tracer also allowed us to follow nitrate to nitrite reduction, as it led to ^15^N-nitrite. As expected, nitrate reductase activity was high in the liver, but skin and bone also showed elevated presences of ^15^N-nitrite. To the best of our knowledge, this is the first study considering the possible role of dietary nitrate as a possible source of NO in the bone and identifying bone and skin as additional reservoirs for storing nitrate.

## 2. Materials and Methods

### 2.1. Animals and Nitrate Supplementation

This research was conducted as part of animal protocols approved by the IACUC (Institutional Animal Care and Use Committee) of Medstar Health Research Institute in compliance with the Animal Welfare Act and the Guide for the Care and Use of Laboratory Animals, 8th ed. [80], and ARRIVE guidelines (Supplementary Materials). Both male and female Yorkshire domestic cross pig weighing between 35 and 65 kg and sourced from Thomas D. Morris, Inc. (Reisterstown, MD, USA) were used. On arrival, animals were acclimated for a minimum of 72 h and housed in an AAALAC (Association for Assessment and Accreditation of Laboratory Animal Care)-accredited facility with environmental enrichment. Animals were fed twice daily with a commercial chow (Teklad miniswine diet, 8753C, Envigo, Madison, WI, USA). Fresh water was provided ad libitum by an automated system. Pigs were randomly divided into 2 groups, using random numbers generated using the standard RAND() function in MS Excel version 2402. The control group and nitrate treatment group consisted each of 2 male and 2 female pigs, *n* = 4, for each group, with a total of 8 animals used for this study. The sample size of the pig groups was determined based on previous reports [81,82] using a resource equation approach suitable for exploratory studies where the effect size and standard deviations are not known. The control group received bolus NaCl, and the treatment groups received Na^15^NO_3_ (1 g/L, ≥98 atom % ^15^N, Sigma, St. Louis, MO, USA, ^15^N-nitrite content less than 0.01%) in one time bolus mixed with a small amount of moist food, 0.15 mmol/kg for placebo, as well as nitrate treatment. Supplementation time was kept constant, and treatment order was randomized daily. Investigators could not be blinded to whether the pigs received placebo or nitrate treatment because all the principal investigators had to participate in the entire process of pig preparation and sample collection due to the large size of the animals.

### 2.2. Sample Collection

On the day of terminal tissue collection, pigs were sedated with a cocktail mixture of ketamine (15–20 mg/kg, Zoetis, Parsippany, NJ, USA) and xylazine (3–5 mg/kg, Covetrus, Dublin, OH, USA) and then maintained under 3–5% isoflurane with 2 L/min oxygen anesthesia with a mechanical ventilator during the entire procedure. Either the left or right common carotid artery and jugular vein was accessed by a 50–75 mm ventral midline neck skin incision, and catheters were inserted. Both the left and right femoral veins were cannulated via ultrasound-guided percutaneous access. Tygon 3350 silicone tubing (Saint-Gobain, MI, USA) was connected to all venous sheaths, and animals were heparinized (≥200 IU/Kg). Arterial and venous blood samples were collected from the carotid artery and jugular vein before heparinization. Blood removal began 15 min after heparin administration with all three venous access lines. Euthanasia was accomplished with a single intravenous injection of saturated potassium chloride (4.6 mL per 10 kg body weight, IV bolus, using a 4.2 M KCl concentration) while the animals were maintained on isoflurane gas anesthesia per the AVMA (American Veterinary Medical Association) guidelines. Animal expiration was confirmed when no visible respirations (apnea) were observed and readings of zero for heart rate, pO_2_ level, and CO_2_ saturation were noted. Perfusion began immediately after euthanasia with the arterial sheath attached to warm 0.9% NaCl solution (Baxter Healthcare, Mississauga, ON, Canada) with heparin (2000 IU/L, Fresenius Kabi, IL, USA) and lasted one hour. Exsanguination and perfusion are necessary steps for protecting nitrite in tissues from reaction with hemoglobin when tissues are homogenized during sample preparation for nitrite determination. A total of 8 L of heparinized 0.9% NaCl solution was perfused before tissue collection from the liver, skeletal muscle (gluteus), mid-section of the femur, skin (front part of scalp), and liver. All samples were placed into microcentrifuge tubes and immediately frozen on dry ice. Samples were stored at −80 °C until analysis. Dissection was carried out simultaneously on different parts of the pigs to speed up the whole process, but samples from a particular pig part were collected always in the same order.

### 2.3. Determination of Total Nitrite and Nitrate Concentrations

Nitrite and nitrate levels in all samples were measured using a standard chemiluminescence method, as described previously [83]. Plasma and urine samples were mixed with cold methanol 1:2 (sample/methanol, vol/vol) for deproteinization and then centrifuged for 30 min (17,000× *g*, 4 °C, AccuSpinMicroR, Fisher Scientific, Pittsburgh, PA, USA). The supernatant was collected, and part of it was further processed for LC-MS/MS (see below). The remaining supernatant was injected into the nitric oxide analyzer (NOA, Sievers, Model 280 i NO analyzer, Boulder, CO, USA) using helium as the carrier gas. Vanadium chloride or tri-iodide solution was used for the nitrate or nitrite analysis, respectively. All other tissue samples were weighed, mixed with water (dilution 1:5 sample/water, vol/vol), and homogenized at 4 C using Precellys Evolution (Bertin Corp, Rockville, MD, USA). Samples were centrifuged at 17,000× *g* for 30 min and the supernatant was collected. At this point, part of the supernatant was processed for LC-MS/MS analysis (see below) and the rest was used for NOA. NOA samples were deproteinized by dilution with cold methanol 1:2 (sample/methanol, vol/vol) and then centrifuged for 30 min (17,000× *g*, 4 °C) to obtain clear supernatant.

### 2.4. Preparation of Samples for LC-MS/MS

To measure the nitrate content by LC-MS/MS, nitrate ions in all samples were first reduced to nitrite enzymatically by bacterial nitrate reductase from Aspergillus niger (N7265, Sigma-Aldrich, St. Louis, MO, USA), as previously described [84,85], with some modification. Briefly, samples (20 µL) were mixed with nitrate reductase (0.1 U/mL) and NADPH (100 µM) and incubated for 2 h at room temperature. Then, nitrite ions in samples were derivatized with 2,3-diaminonaphthalene (DAN, D2757, Sigma-Aldrich) for 30 min at 37 °C to yield 2,3-naphthotriazole (NAT). NaOH (58 mM) was added to terminate the reaction. For measuring the nitrite content only, samples (50 µL) were directly subjected to DAN derivatization.

### 2.5. Determination of ^15^N-Nitrate or ^15^N-Nitrite Percent by LC-MS/MS

High-performance liquid chromatography (HPLC)-grade solvents and LC-MS modifiers were purchased from Sigma-Aldrich (St. Louis, MO, USA). Detection and quantification were achieved by ultra-performance liquid chromatography–tandem mass spectrometry (UPLC-MS/MS) utilizing a Thermo Scientific Vanquish UPLC with a Thermo Scientific Altis triple quadrupole mass spectrometer and heated electrospray ionization (HESI-II) in positive-ion mode (3500 V) (Thermo Fisher Scientific, Germering, Germany). In total, 50 µL of sample was mixed with 200 µL of acetonitrile (ACN), vortexed for 5 min, and then centrifuged at 4 °C, 17,000× *g* for 15 min. The supernatant was transferred to an LC-MS vial for analysis. The injection volume was 1 µL. A Waters Cortecs T3 column, 2.1 × 100 mm and 1.6 µm, was maintained at 35 °C. Solvent A: H_2_O with 0.1% formic acid (FA) and Solvent B: ACN with 0.1% FA. The flow rate was 250 µL/min, the gradient was 25% B at 0 min for 0.25 min, increasing to 65% B at 5 min, further increasing to 90% B at 5.5 min, remaining at 90% B until 7.5 min, and then decreasing to 25% B at 8 min. The total running time was 10 min. Samples were analyzed in triplicate. The quantitation of ^14^NAT and ^15^NAT was based on multiple reaction monitoring (MRM) transitions *m/z*, 170.062 → 115.042 and 171.062 → 115.042, respectively. The result was based on the percentage ratio of ^15^NAT/(^14^NAT + ^15^NAT).

### 2.6. Statistical Analysis

Values represent the average ± standard deviation. The statistical significance of the results was tested using a one-way ANOVA. * denotes *p* < 0.05.

## 3. Results

Previously, we showed [44] that when compared to its levels in plasma and liver, skeletal muscle contains higher concentrations of nitrate. Here, we explore further the possibility of the existence of other tissues/organs in mammals able to concentrate nitrate ions for further use.

To distinguish between nitrate previously stored in tissues and “newly incoming” nitrate, we used ^15^N-labeled nitrate for acute nitrate supplementation. ^15^N isotope is a stable naturally occurring nitrogen isotope with a relative abundance of 0.4% [86].

### 3.1. Nitrate

**Figure 1:**
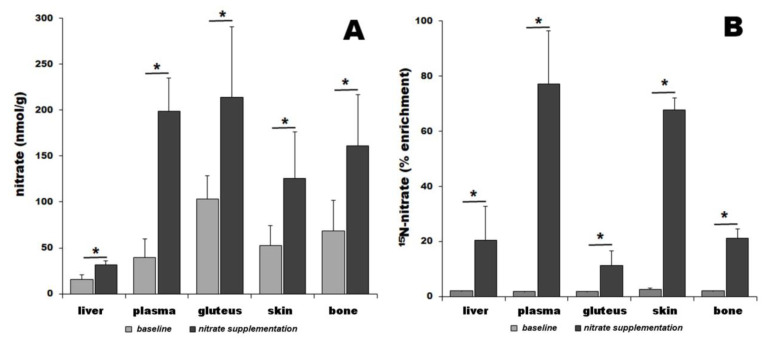
Total nitrate in pig organs determined by chemiluminescence (**A**) and organ enrichment by ^15^N-labeled dietary nitrate (**B**). Nitrate levels were measured at baseline (gray bars) and 3 h after ^15^N-nitrate supplementation (black bars). Star “*” denotes *p* < 0.05; number of animals in each treatment group, *n* = 4.

Nitrate is not distributed homogenously among organs at baseline conditions, but there is a distinct nitrate concentration gradient, with the highest concentration occurring in skeletal muscle (gluteus, 103.5 ± 24.6 nmol/g tissue), followed by bone (68.4 ± 33.1 nmol/g), skin (52.6 ± 21.4 nmol/g), plasma (39.8 ± 19.7 nmol/g), and liver (15.7 ± 5.2 nmol/g)—Figure 1A, baseline values, gray bars. Relative comparison at the baseline level shows 2.6-fold, 6.6-fold, 4.3-fold, and 3.3-fold higher nitrate content in plasma, gluteus, bone, and skin than in liver, respectively. Three hours after animals are fed ^15^N-labeled nitrate, a significant nonuniform increase in total nitrate levels in all tissues is observed compared to its baseline levels—plasma (5-fold, 199.0 ± 35.6 nmol/g), bone (2.4-fold, 161.2 ± 55.6 nmol/g), skin (2.4-fold, 125.9 ± 50.1 nmol/g), gluteus (2.1-fold, 213.8 ± 77.0 nmol/g), and liver (2-fold, 31.2 ± 4.9 nmol/g)—Figure 1A, black bars. Relative comparisons after supplementation show 6.4-fold, 6.8-fold, 5.2-fold, and 4.0-fold higher nitrate content in plasma, gluteus, bone, and skin than in liver, respectively. We also analyzed samples using mass spectrometry to determine the fraction of ^15^N-nitrate in total nitrate at baseline (Figure 1B, light gray bars) and three hours after ^15^N-nitrate bolus (Figure 1B, black bars). At baseline, ^15^ the N-nitrate level was constant in all organs at 2.1 ± 0.3%. After dietary intervention, ^15^ the N-nitrate levels in all organs increased significantly and in a nonuniform way: 77.1 ± 19.1% of total nitrate in plasma was ^15^N-nitrate, followed by scalp skin with 67.7 ± 4.3%, femur 21.0 ± 3.5%, liver 20.4 ± 12.4%, and gluteus 11.4 ± 5.3%.

### 3.2. Nitrite

**Figure 2:**
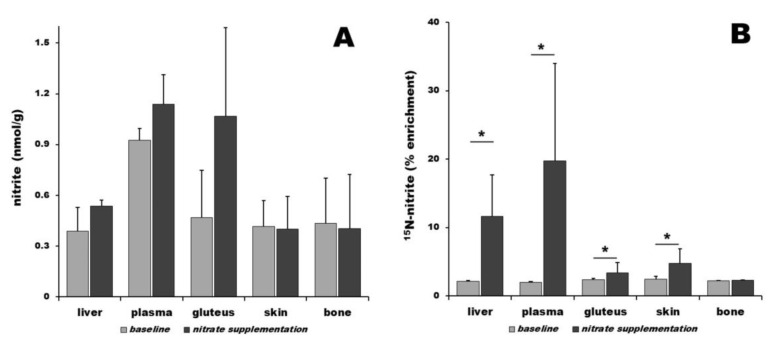
Total nitrite in pig organs determined by chemiluminescence (**A**) and organ enrichment by ^15^N-labeled nitrite (**B**). Nitrite levels were measured at baseline (gray bars) and 3 h after ^15^N-nitrate supplementation (black bars). Star “*” denotes *p* < 0.05; number of animals in each treatment group, *n* = 4.

Nitrite distribution among organs at the baseline conditions is nonuniform, with plasma containing 0.93 ± 0.07 nmol/g of nitrite, gluteus 0.47 ± 0.28 nmol/g, bone 0.43 ± 0.27 nmol/g, skin 0.42 ± 0.15 nmol/g, and liver 0.39 ± 0.14 nmol/g—Figure 2A, gray bars. Relative comparison at baseline level shows 2.4-fold, 1.2-fold, 1.1-fold, and 1.1-fold higher nitrite content in plasma, gluteus, bone, and skin than in liver, respectively. Three hours after ^15^N-nitrate ingestion, there is a nonsignificant increase in nitrite in gluteus (2.3-fold, 1.07 ± 0.52 nmol/g), plasma (1.2-fold, 1.14 ± 0.17 nmol/g), and liver (1.4-fold, 0.54 ± 0.04 nmol/g). Nitrite levels in skin and bone remained the same as at baseline level, as shown in Figure 2A, black bars. Relative comparison after supplementation shows 2.1-fold and 2.0-fold higher nitrate contents in plasma and gluteus than in liver, respectively. Mass spectrometry was used to determine the fraction of ^15^N-nitrite in all samples—Figure 2B. At baseline, the ^15^N-nitrite level was constant in all organs at 2.3 ± 0.2%. After dietary intervention, the ^15^N-nitrite levels in all organs (except bone) increased significantly and in a nonuniform way: 19.8 ± 14.2% of total nitrate in plasma was ^15^N-nitrite, followed by liver with 11.6 ± 6.1%, 4.8 ± 2.1% in skin, 3.4 ± 1.6% in gluteus, and 2.3 ± 0.04% in bone.

## 4. Discussion

When the nitrate reductive pathway in oral bacteria leading to nitrite and NO was discovered, it was thought that mammals do not possess native nitrate reductases in their cells. The original idea was that (dietary) nitrate is reduced into nitrite by salivary bacteria nitrate reductases [15,87,88], swallowed and processed into NO by the acid in the stomach [89] or in tissues by nitrite reductases [9,12]. Later, the nitrate active transporter, sialin, was identified in salivary glands [90], and it was also shown that sialin is expressed in almost all tissues [91,92,93]. This hypothesis of the importance of nitrate stores does not consider the possibility of an internal nitrate pool in the mammalian body, as it presumes that most of the nitrate is coming regularly from dietary sources or oxidation of NO (from NOS-related and reductive pathways) and nitrite back to nitrate by physiological processes [94,95,96].

However, since NO is crucial for maintaining several vital physiological cycles, among them, proper blood flow, and tissue perfusion, we speculated about the existence of internal nitrate pools that would serve as a backup system in the case of low dietary nitrate availability and/or impaired NOS activity. We suggested two main requirements of the ideal organ/tissue to serve as a nitrate reservoir: a large organ and relatively low metabolic activity most of the time. The skeletal muscle is one of the largest organs in the mammalian body and, with some exceptions, is almost never engaged entirely. In addition, it also requires efficient in situ blood flow regulation, and during its functioning (exercise), general tissue oxygenation can reach low levels [97]. All this suggested to us that the skeletal muscle is the prime candidate for the function of the nitrate reservoir. Data confirmed elevated nitrate concentrations in skeletal muscles from rodents, pigs, and humans compared to plasma and other internal organs, including liver at baseline conditions [38,44,45,98,99,100,101]. Later, using dietary nitrate supplementation and ^15^N-labeled nitrate, it was established that the nitrate reservoir in skeletal muscle is easily accessible to nitrate fluxes in and out of the muscle [49,51].

In addition to the skeletal muscle, skin and skeleton (bone) are two other large organs with relatively slower metabolism, at least at resting conditions. We hypothesized that these two organs are also good candidates for the function of the nitrate reservoir. Using the pig as an animal of choice, due to its similarities with humans in physiology and metabolism, we measured nitrate and nitrite in different organs at baseline conditions and three hours after the ingestion of ^15^N-labeled nitrate. Indeed, baseline measurements of total nitrate levels in bone, skin, and gluteus showed several-fold increases in its content when compared to plasma and liver (Figure 1A and Figure 2A). This supports the idea that these tissues all function as internal nitrate pools/reservoirs, similarly to the already-shown nitrate reservoir in skeletal muscle [44]. Besides being conveniently large, organs with relatively slower metabolism (when compared to highly active organs such as liver, heart, or brain), skeletal muscle, and skin could also benefit from the locally stored nitrate directly in situ. Both these organs are at some point needed to increase local blood flow rather rapidly; the skeletal muscle, when an action requiring rapid blood flow increase is needed (heavy exercise, fleeing from a predator, etc.) [97], and the skin, when excessive heat dissipation is required, caused by external (exercise, rising external T) or internal (fever) stimuli [102,103,104,105,106,107]. If needed, skin blood flow can increase up to 7-fold from its resting state [105]. In addition to thermoregulation via blood flow changes, skin also functions as a barrier and protection from the external world, especially from any type of pathogenic microorganisms. Skin is also frequently the site of minor injuries, such as abrasions and cuts that could potentially become sites of pathogens’ entry into the body. NO is a potent antimicrobial agent and part of body immune responses to pathogens [108,109,110]. In addition, NO also promotes angiogenesis, which is important for the recovery of the skin barrier in the case of larger injuries [111,112]. Therefore, it should not become such a surprise that skin tends to sequester and store nitrate from the blood [65].

It is known that the skeletal muscle possesses the enzymatic equipment necessary to reduce nitrate into nitrite and NO in the form of xanthine oxidoreductase (XOR, nitrate/nitrite reductase) and myoglobin/other heme proteins as nitrite reductase. Even with the low nitrate reductase activity of XOR, one should consider the large volume of muscle tissue involved, high nitrate concentrations in it, and the low final quantities of NO required for action. The contribution from NOS pathways, albeit diminishing with decreasing oxygen levels, should also be considered, especially at the beginning of exercise when oxygen is still relatively available. Also, with the size of the effect still not evaluated, the changes in the contribution of salivary bacteria into plasma nitrite with exercise could play a supportive role by making more nitrite in skeletal muscle available for nitrite reductases.

Vis a vis the idea of nitrate reservoirs, skin bears a striking resemblance to skeletal muscle. Being a large organ with an abundant vascular bed, the skin consists of several layers with different levels of metabolic activity. We did not separate different skin layers for our measurements, so our data reflect the average nitrate concentration through all layers. Similar to skeletal muscle, there is a requirement for a rapid increase in blood flow to the skin as a whole or to some parts of the skin, especially when heat dissipation is needed. So, one can apply the same reasoning as already explained for skeletal muscle about the advantage of storing excess body nitrate as the skin nitrate reservoir. An additional advantage when compared with muscle is that this nitrate pool is likely accessible to symbiotic skin bacteria, with some of them being able to reduce nitrate into nitrite that could then diffuse back to the skin and become reduced to NO. This process could also be facilitated by the fact that when heat dissipation is needed, sweat is excreted to the surface of the skin where bacteria reside [53], and the moist skin could also facilitate the diffusion of nitrite back to the body. However, these hypotheses still need to be explored in more detail.

We find the most intriguing finding to be the high nitrate concentration in the skeleton itself. We believe that this could be the largest reservoir or “deposit” site, where nitrate would need to be eluted into the blood and transported into other tissues for reduction to nitrite and NO. Nitrate could be embedded into the mineral part of the bone, perhaps becoming the “last resort” when all other pools of stored nitrate are depleted. However, this idea also needs to be explored in more detail. Interestingly, it had been demonstrated that NO has role in bone healing [113] and affects blood flow in the bone tissue [70].

The second part of our study traces the short-term mobility of dietary nitrate. The use of ^15^N-labeled nitrate allowed us to look at the distribution of ingested nitrate 3 h after the administration of a bolus, a timepoint that coincides with the peak nitrate levels in blood and other tissues. We found that all tissues are eagerly accepting the “new” nitrate provided by diet. Relative to their baseline levels, the nitrate enrichment varied from 77% in plasma to 68% in skin, 21% in bone, 20% in liver, and 11% in gluteus. This statement translates as 77% of all nitrate in plasma being ^15^N-nitrate, and so on. High levels of ingested nitrate in plasma are not surprising, as bloodstream is the distribution route of all ingested nutrients. What is somehow unexpected, but not so surprising, is the quick rise of nitrate levels in the skin (enrichment 64%) that almost reaches nitrate enrichment in plasma. Skin is the largest organ of the body with substantial vascular beds, containing about ~38 cm/cm^2^ of blood vessels [114]. While skeletal muscle is also highly vascularized tissue, we do only see 11% enrichment by ^15^N-nitrate, which is a bit surprising, but the explanation for this might well be the fact that after the administration of bolus nitrate, pigs were not exercising, and therefore their muscles have only baseline blood flow with less blood flowing through them than in the skin, so ^15^N-nitrate was somehow less accessible at that time frame. Also, one should keep in mind that skeletal muscle is the largest organ and, therefore, the total amount of nitrate stored in it, albeit at a slower absorption rate, is still very substantial. However, the most surprising fact is that the enrichment of nitrate in the skeleton by ^15^N-nitrate reached 21%. We believe that this shows well that bone tissue is actively sequestering nitrate from the blood and storing it in the tissue. In agreement with its role as the main nitrate processing site by mammalian nitrate reductases (XORs), nitrate enrichment in the liver reaches 20%.

In contrast to nitrate, at baseline levels, nitrite is distributed mostly uniformly across organs, with the exception of plasma containing a somehow elevated concentration of nitrite. Three hours after ^15^N-nitrate distribution, there are no significant changes in nitrite levels compared to baseline in any organs, except in skeletal muscle, where the nitrite concentration doubles, but this difference does not reach a level of statistical significance. Three hours after ^15^N-nitrate bolus is administered, one can see a significant elevation in ^15^N-nitrite in all organs, except bone, compared to their respective baseline levels. The highest levels of ^15^N-nitrite, 20%, is found in plasma, reflecting the combination of salivary bacteria ^15^N-nitrate to ^15^N-nitrite reduction and the ^15^N-nitrate reductase activity of the liver, where 12% of total nitrite is ^15^N-nitrite. There is a small but significant increase in ^15^N-nitrite in the gluteus (3.4%) and skin (5%). We believe that this situation reflects the fact that liver is the main site of mammalian nitrate reduction into nitrite, with other organs being mainly its “recipients”, with only marginal nitrate reduction in situ, especially in the non-exercising state.

**Figure 3:**
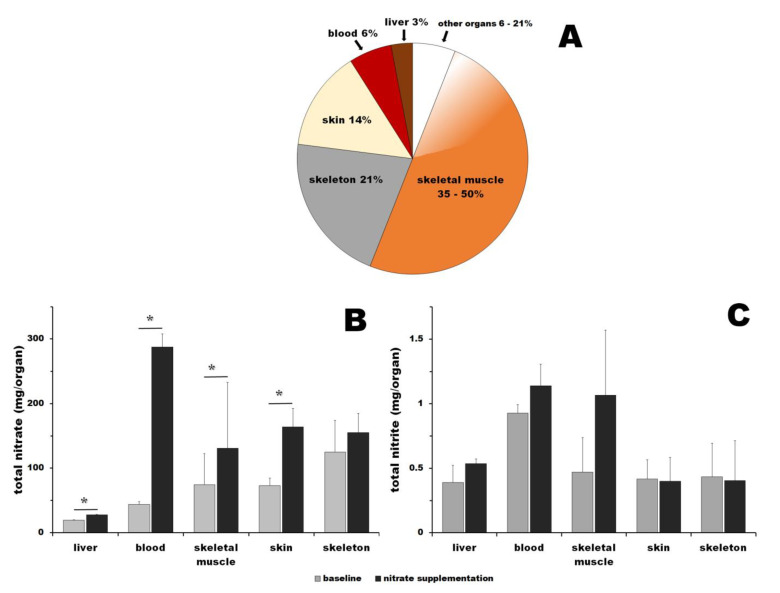
(**A**) Relative weight contribution of various organs to total body weight for 70 kg male human (compiled from values [117,118,119,120,121,122,123]). (**B**) Estimation of total nitrate stored in 70 kg body based on nitrate concentrations determined for pig in this study and organ/tissue weights from panel (**A**) for human. Depending on the skeletal muscle mass, there could be a total of 366–523 mg of nitrate at baseline conditions and 952–1340 mg of nitrate 3 h after nitrate bolus ingestion stored in the human body based on the body skeletal muscle proportion. (**C**) Estimation of total nitrite stored in 70 kg body based on nitrate concentrations determined for pig in this study and organ/tissue weights from panel (**A**). Depending on the skeletal muscle mass, there could be a total of 2.5–3.6 mg of nitrite at baseline conditions and 3.4–4.9 mg of nitrite 3 h after nitrate bolus ingestion stored in the human body based on the body skeletal muscle proportion. Star “*” denotes *p* < 0.05; values for skeletal muscle on (**B**,**C**) presume body composition with 35% skeletal muscle.

NO is necessary for many healthy functions in mammals. Studies on model animals and humans have associated low NO availability with various diseases and states, such as metabolic syndrome, diabetes, muscular dystrophias, endothelial dysfunction, and even some forms of dementia. In addition to the canonical NO formation by NOS, a concurrent reductive pathway of nitrate reduction to nitrite and NO supplies NO. This pathway becomes predominant at limited oxygen availability. Nitrate is supplied mainly by diet, vegetables being the richest provider of this ion. Since, historically, fresh vegetables were only seasonally available, we suspected that nitrate might be stored in the mammalian bodies for compensating the seasonal availability of this resource. First, we formulated several requirements for a good nitrate storage organ/tissue, such as a sufficiently large organ with relatively low metabolism, conditions that allow for nitrate concentrations to remain low enough not to interfere with physiological processes while temporarily raising the presence of charged ions (NO_3_^−^). Skeletal muscle was identified as a prime candidate due to its large mass (Figure 3A) and pronounced functional hyperemia [97,115,116] that leads to increased NO requirements at periods of time. When tested, we confirmed that skeletal muscle tissue is a nitrate reservoir and, using dietary nitrate manipulations, we showed that skeletal muscle is not only a nitrate reservoir but that nitrate reduction also occurs in this tissue itself [52].

When the abovementioned criteria for effective nitrate storage requirements are considered, two other possible candidates emerge—skin and skeleton (Figure 3A). Neither of them were previously considered as possible nitrate storage sites. In the current study, we present the first evidence that both of these organs contain a nitrate pool that responds to changes in nitrate availability from the outside—mainly diet. These reservoirs are easily accessible via the bloodstream. Further studies are needed to work out the relative importance and dynamics of nitrate flows in/out and between these reservoirs, as well as the benefits of high nitrate consumption for general well-being and as supporting therapies for NO deficiency states. To estimate the amount of nitrate that could be stored in nitrate reservoirs in each organ and its total amount at baseline and after nitrate supplementation, we combined available data about the size of human organs for a 70 kg human male [117,118,119,120,121,122,123] with nitrate and nitrite concentrations in organs determined in this study for pig. Unfortunately, for humans, direct nitrate and nitrite concentrations are widely known mainly for blood and its components [10,11,13,14,26,32,33,34,45,50,51] and several measurements from skeletal muscle biopsies [45,50,51,98,99,100], making it impossible to estimate these numbers directly. However, nitrate/nitrite concentrations in human skeletal muscle and blood from our previously published studies on humans [45,50,51] are in reasonable agreement with our present pig data. Therefore, we took the liberty to combine these two sets of data and try to make a rough estimation of the total content of nitrate and nitrite that could be contained and stored in the human body. Depending on the skeletal muscle mass for a 70 kg human male (35–50%), the estimation leads to a total of 366–523 mg of nitrate at baseline and 952–1340 mg after supplementation, which is a 2.6-fold increase from baseline (Figure 3B). The same calculations for nitrite lead to estimations of a total of 2.5–3.6 mg of nitrite at baseline conditions and 3.4–4.9 mg of nitrite 3 h after nitrate bolus ingestion, which represents a 1.4-fold increase from the baseline (Figure 3C). Interestingly, it is still unknown what the “normal” levels of nitrate/nitrite are for humans, and it is quite possible that most modern humans, especially in the Western hemisphere, are deficient in these two ions. Epidemiological studies of different populations worldwide, still living in traditional ways in various geographical regions and consuming diverse diets, could give insights into the desirable levels of these micronutrients. We believe that such knowledge could lead to significant revisions of current dietary recommendations and improvements in health, especially in terms of the cardiovascular system.

## 5. Conclusions

In mammals, the nitrate reductive pathway supplies a significant portion of NO. This pathway is, for a great part, fueled by dietary nitrate. Historically, the availability of green leafy vegetables, the main source of dietary nitrate, was only seasonal. We hypothesize that the mammalian body evolved to store enough nitrate for times of its deficiency. We formulate three criteria for adequate nitrate reservoir/storage organs: a large volume with relatively low metabolism and well-regulated blood flow. These conditions allow for nitrate concentrations to remain reasonably low and not to disrupt physiological processes due to the presence of higher concentrations of ions, while assuring rapid access to the reservoir by temporary increasing blood flow into organs. According to these standards, skeletal muscle, skeleton, and skin emerged as prime candidates for the role of nitrate reservoirs. In addition to our previous evidence of skeletal muscle as a nitrate reservoir, here we confirm that the skeleton and skin are also important nitrate reservoirs in the mammalian body.

## Data Availability

The raw data supporting the conclusions of this article will be made available by the corresponding author upon reasonable request due to an agreement with the NIH practice.

## Supplementary Materials

The following supporting information can be downloaded at: https://www.mdpi.com/article/10.3390/nu16162674/s1, The ARRIVE guidelines 2.0: author checklist.
